# Promoting development and uptake of health innovations: The Nose to Tail Tool

**DOI:** 10.12688/f1000research.8145.1

**Published:** 2016-03-16

**Authors:** Archna Gupta, Cathy Thorpe, Onil Bhattacharyya, Merrick Zwarenstein

**Affiliations:** 1 Centre for Studies in Family Medicine, Department of Family Medicine, Schulich School of Medicine & Dentistry, Western Centre for Public Health and Family Medicine, Western University, London, ON, Canada; 2 Women's College Hospital, Toronto, ON, Canada

**Keywords:** Health innovation, pilot test, implementation, scale up, stakeholders, researchers, end users, decision makers

## Abstract

**Introduction**

Health sector management is increasingly complex as new health technologies, treatments, and innovative service delivery strategies are developed. Many of these innovations are implemented prematurely, or fail to be implemented at scale, resulting in substantial wasted resources.

**Methods**

A scoping review was conducted to identify articles that described the scale up process conceptually or that described an instance in which a healthcare innovation was scaled up. We define scale up as the expansion and extension of delivery or access to an innovation for all end users in a jurisdiction who will benefit from it.

**Results**

Sixty nine articles were eligible for review. Frequently described stages in the innovation process and contextual issues that influence progress through each stage were mapped. 16 stages were identified: 12 deliberation and 4 action stages. Included papers suggest that innovations progress through stages of maturity and the uptake of innovation depends on the innovation aligning with the interests of 3 critical stakeholder groups (innovators, end users and the decision makers) and is also influenced by 3 broader contexts (social and physical environment, the health system, and the regulatory, political and economic environment). The 16 stages form the rows of the Nose to Tail Tool (NTT) grid and the 6 contingency factors form columns. The resulting stage-by-issue grid consists of 72 cells, each populated with cell-specific questions, prompts and considerations from the reviewed literature.

**Conclusion**

We offer a tool that helps stakeholders identify the stage of maturity of their innovation, helps facilitate deliberative discussions on the key considerations for each major stakeholder group and the major contextual barriers that the innovation faces. We believe the NTT will help to identify potential problems that the innovation will face and facilitates early modification, before large investments are made in a potentially flawed solution.

## Introduction

While innovation in drugs, technologies, procedures and healthcare delivery approaches is a major influence on health systems, uncertainty around their benefits and unintended consequences complicates the management of innovation in the healthcare system. While innovation is almost universally attempted the vast majority of health innovation ideas do not progress into viable products, services or changes in healthcare delivery. Few of those that are successfully developed and pilot tested in one locale are implemented effectively or achieve expected outcomes in that initial locale, and even fewer scale up to their full potential, eventually to be institutionalized into common practice: fewer than 5 percent of drug or technology innovations reach scale and are sustained ^1^. And yet, this low success rate for scaling up a new health intervention takes on average 14 years and 2 billion US dollars per successful effort; the cost of the unsuccessful efforts is unknown and not included. The proportion of successes is simply not known for health service delivery or policy changes, nor is the cost known. Given the relatively low investment in early stage development and evaluation of healthcare delivery innovations, and the absence of a regulatory framework judging the balance of benefits, cost and harm, the success rate may be even lower.

Analyses of unsuccessful efforts to scale up innovations provides insights into why scale up is rare ^2^. Common challenges include underestimating the resources required for scale up, failure to understand the importance of politics and policy in successful scale up, not considering the conditions needed for scale up early in the process of innovation development and an overemphasis of either the vertical or horizontal spread of innovations as opposed to considering both ^2^. This relatively simple set of causes is belied by the chaos of the theoretical literature on the same topic. A recent review of models and frameworks for dissemination and implementation found 61 such frameworks ^3, 4^, many overlapping conceptually, but with no common terminology. A shared terminology would improve communication among and between researchers and implementation groups ^3^.

Based on the apparent simplicity of the problems inhibiting successful scale up, and doubting the value of yet another theoretical framework, we elected to take a different approach to the problem of improving success in the scale up of innovations ^5^. In this paper we describe an atheoretical, stage based tool that was developed for stakeholders, who may be developing, testing, implementing, funding or regulating a particular innovation. The tool helps stakeholders identify the stage of maturity of their innovation and helps facilitate deliberative discussions on the key considerations for major stakeholder groups and the major contextual barriers that the innovation faces. The goal is to help innovation teams identify which issues have been successfully managed up to their present stage of development and identify issues that still need to be addressed to move the innovation forward towards scale up. The tool incorporates research papers from several “disciplines”, such as rapid cycle innovation, dissemination and implementation science, knowledge translation and quality improvement that currently study innovation development and deployment. We merge ideas from all of these into an overarching tool that goes from nose (the problem and the initial idea for its solution) to tail (scale up and sustaining the solution) of the innovation process.

### The Nose to Tail Tool

The Nose to Tail Tool (NTT) is intended to offer innovation teams, consisting of innovators and the essential stakeholders including end users and decision makers, a guide to: (a) identify what stage in the process their idea/innovation is at; (b) identify key considerations from each stakeholder perspective that should be addressed at the stage that their innovation has reached; and (c) identify contextual barriers at that stage that may be fatal to an innovation’s success and which must be overcome to move forward. The NTT is a comprehensive and consistent way of prompting context and stakeholder aware planning through the entire innovation process. The tool was designed with the belief that if stakeholders of an innovation consider the end stages from the very beginning of the process their innovation is more likely to achieve scale up and institutionalization ^2, 6, 7^. This tool helps innovation teams identify barriers, both those that can be overcome, and those that cannot, early in the innovation process, giving teams an opportunity to re-design the innovation at an early stage, or the opportunity to cease work on the project before too many resources have been invested.

The tool is aimed at newly developed innovations in healthcare and it is assumed that innovators have already excluded the availability of an existing intervention, either from the health field or products in another field that could potentially address the problem. The tool however can also be used to adapt existing innovations, developed elsewhere or for another situation or problem, into a different context.

Health innovations can be described as discrete innovations, multicomponent interventions and paradigmatic innovations ^2^. Discrete innovations are simple and well defined such as zinc in early childhood ^8^, combination therapy ART ^9^ or the use of new technology for diagnosis and treatment of TB ^10^. Multicomponent interventions involve several interacting program elements to produce a composite set of innovations that may also be targeted at multiple system levels ^2^. Examples include multilevel initiatives to decrease childhood obesity ^11^ or scale up of post abortion care services ^12^. Paradigmatic innovations require a shift in the way we understand health problems and the potential solutions to address them ^2^. China’s quality of care reforms for family planning, is an example, which required a systems wide approach, and partnerships between international groups and all levels of governments in China, including those that extend outside of public health ^13^. Paradigmatic innovations attempt to address the causes of poor health by using a determinants of health approach; they are complex, require a systems level approach and partnerships among key stakeholders from across sectors ^2^. Given their size and complexity, paradigmatic innovations are more difficult to stage and assess feasibility; however, in many cases they can be broken down into several smaller components similar to individual discrete or multicomponent innovations working together. The NTT may be most usefully applied to simple and multicomponent innovations; including service delivery, diagnostic, product, device or information technology innovations.

## Methodology

We conducted a scoping review of the literature to identify whether a pre-existing dialogue or tool existed that could be used to help innovators and decision makers successfully implement and scale up innovations ^14^. Through this review it was identified that such a tool did not exist.

The initial search terms used for the scoping review were duplicated from Yamey’s article, “Scaling Up Global Health Interventions: A proposed Framework for Success.” ^15^ The search terms “global health” in text or “international health” in text and “implementation science” in title [ti] and abstract [ab] or “scaling up” in title or “scaling-up” in title were used in PubMed on June 19, 2013. Non-English articles were excluded. This search resulted in 13 full text articles. The titles and abstracts for these articles were reviewed by MZ and 5 articles were selected. Articles were selected if they described a process whereby an innovation was scaled up or if authors conceptually described the scale up process. We were specifically interested in papers that highlighted the process of jurisdiction or organization wide scale up. To broaden the review for global coverage, a second search was done in PubMed excluding the terms “global health” and “international health, leaving “implementation science[tiab] OR scaling up[ti] OR scaling-up[ti]”. This search strategy retrieved 383 full text articles. The titles and abstracts for these articles were reviewed by MZ and 60 additional articles from this second set were deemed relevant for a total of 65 articles. Articles were then read in full and validated by AG and two articles were excluded. Additional articles (n=6) were then included from the reference lists of papers identified in PubMed searches, from key informants in the field, and from the investigator’s own files. In total 69 articles were included. Over half of the articles reviewed (n=35) come from literature based in or describing innovations in the low and middle income country (LMIC) context.

The scoping review was not meant to be an extensive literature review, but rather a means to identify, using the qualitative research concept of saturation, enough relevant studies to describe a coherent set of stages of the innovation process and the major considerations for each stage. Key information from the literature was sorted and charted according to the key issues and themes using a narrative review approach. All articles were printed and read in full with key phrases and sections typed manually into an Excel spreadsheet ( Dataset 1). Information was recorded under the following headings: Authors, Title, Summary and Purpose, Referenced Papers and Theories, Stages Discussed, Methods Used, Research Values, and Details of the Stages Discussed. The information was then collated and summarized paying attention to the frequency in which ideas appeared. The literature was reviewed until saturation was reached. The scoping review was conducted using a realist approach utilizing a heterogeneous collection of studies including primary papers as well as reviews; papers were included if they described a process whereby an innovation was scaled up or conceptually described the scale up process; data extraction and analysis used an iterative approach; and an iterative approach was also used to extract and analyze data ^16^.

The included articles from the search (n=63) were reviewed by AG to define and map frequently described stages in the innovation process and themes that influence success in these stages. The articles were reviewed again to identify contingency factors and important considerations that should be asked at each stage. These contingency factors were sub-categorized under the two broad themes.

Development of the tool, including stages, definitions and contingency factors was an iterative process. In the first iteration AG reviewed the articles and proposed a number of stages. After discussion with MZ and CT, stages were agreed on, which were then validated against the included articles. We repeated this process for both themes and then contingency factors. Each of the three categorizations (stages, themes and contingency factors) went through three iterations. After these three iterations, we reached the point where the stages and contingency factors were well defined and mutually exclusive. We regarded this process as complete when AG and MZ could use the tool independently to categorize both stage and contingency factors for a large group of innovations in the same way. From these 63 articles we successfully built a grid with series of rows (stages) and columns (contingency factors) resulting in 72 cells, into which content from these articles were added and edited to avoid duplication within each cell. This text was reframed in the form of asking questions to the user of the tool. When we completed the extraction of information from the 63 articles we felt some cells were insufficiently detailed so we then snowballed from the reference lists of the included papers and sought suggestions for other papers from authors and experts. An additional 6 articles were included to increase content in these identified cells. At this point we agreed that each cell had sufficient coverage. Finally, the tool was compared by AG to other frameworks and tools that were described in the literature reviewed to identify similarities and differences.

## Results

NTT scoping review Includes the initial information collected from the initial 63 articles reviewed which were used to determine the stages (rows) and contingency factors (columns) for the NTT. Click here for additional data file. Copyright: © 2016 Gupta A et al. 2016 Data associated with the article are available under the terms of the Creative Commons Zero "No rights reserved" data waiver (CC0 1.0 Public domain dedication).

### Development of the NTT

**Figure 1.**
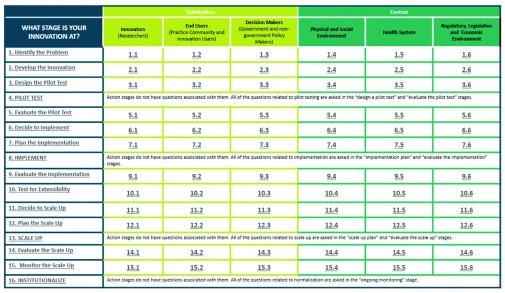
Image of the NTT Grid.

From the primary literature we identified sixteen commonly described stages of innovation development: (1) identify the problem ^12, 17– 24^, (2) develop the innovation ^15, 21, 22, 24– 31^; (3) design the pilot test ^17, 22, 29– 31^; (4) pilot test; (5) evaluate the pilot test ^13, 22, 24– 26, 31– 34^; (6) decide to implement ^12, 15, 17, 19, 21, 22, 28, 35– 41^; (7) plan the implementation ^15, 17, 21– 25, 27, 28, 33, 35– 40, 42– 49^; (8) implement; (9) evaluate the implementation ^22– 27, 31, 32, 42, 48, 50– 53^; (10) test for extensibility ^25, 26, 31, 51, 53, 54^; (11) decide to scale up ^12, 18, 20, 52, 53, 55– 59^; (12) plan the scale up ^12, 13, 20, 27, 31, 33, 34, 36, 42, 47, 52, 55– 67^; (13) scale up; (14) evaluate the scale up ^11, 18, 31, 42, 52, 68^; (15) monitor the scale up ^17, 36, 56, 68^; and (16) institutionalize ^12, 13, 42, 47^. These sixteen stages are used as the rows of the NTT grid ( Figure 1). Of the sixteen stages 12 are considered deliberation stages (1–3, 5–7, 9–13, 14–16) and 4 are action stages (4,8,13,16). Stages 3, 7, 12 and 15 are considered design and planning stages which prepare innovation teams for the action stages 4, 8, 13 and 16. Stages 5, 9, and 14 are evaluation stages. Stages 6 and 11 are unique in that they are decision stages that encourage innovation teams to consider critically whether they are ready for implementation or scale up.

Stage 10, testing for extensibility, is unique because the concept of the stage was acquired by the scoping review, however nowhere in the literature was it defined. It was typically described as an undertaking that would aid with successful scale up, however given its significance, we defined it as a stage in itself. Extensibility in the NTT defines the stage where innovation teams should “conduct multiple studies in various settings and with variable populations” to ensure that the innovation can produce positive outcomes in contextually different, or heterogeneous, environments ^54^. If through testing it is shown that the innovation is no longer as effective, innovation teams should try modifying adaptable components of the innovation. Many interventions are complex and can be conceptualized as being composed of core essential components that cannot be altered without harming integrity and adaptable components that can be altered to fit context ^25, 31^. In our model, it is during the first 5 stages that innovation teams should be preparing for future extensibility; considering the need for future adjustment, adaption or growth. The term “extensibility” is borrowed from the field of software and systems engineering which describes it as “the ability of a system to be extended with new functionality with minimal or no effects on its internal structure” ^69^ or the “capability to adjust and adopt to a variety of reporting [or environment] demands” ^70^.

Scale up is most commonly described as the general process of increasing or spreading coverage of health interventions. However, given that the reviewed literature proposes that testing for extensibility is a facilitator of successful scale up, or a pre-requisite, we propose that scale up requires two steps; spread to first similar and second to different settings. This spread can be to more units, patients, facilities or settings that are rather similar to those in which initial implementation took place which we name as *expansion*, or spread to sites which may be different, which we name as *extension*. While presently there is no distinguishing use of the term expansion in knowledge translation we propose its use to mean spread to homogenous sites, contrasting nicely with extension, and its attendant meaning of stretching.

In addition to the literature suggesting that innovations progress through several stages on route to scale up and institutionalization, it also suggests that the ability to progress forward is contingent on several factors. It is dependent on the characteristics of the innovation itself ^21, 23– 25, 29– 31, 33, 34, 36– 38, 40, 42, 53, 55, 57, 62, 68, 71, 72^ and the interests of the key stakeholders including: a) innovators (researchers) who are involved in developing the innovation ^11, 15, 20, 22– 25, 27– 34, 37, 41– 44, 48, 52, 53, 56^; b) end users (the practice community and innovation users) from the health system unit (i.e. organization, clinic, hospital, community, province etc.) or patients ^11– 13, 18, 20– 25, 27, 29– 31, 33– 39, 41– 43, 47, 48, 53, 56, 62, 65, 72^; and c) decision makers (government and non-government policy makers) who have policy jurisdiction within the health system unit ^12, 13, 15, 18– 20, 25, 28, 32, 34, 36, 37, 40, 42, 43, 48, 53, 61, 62, 67, 72^. It is also dependent on the broader context including the social and physical environment ^11, 15, 17– 19, 22– 25, 28, 31, 34, 40, 53, 56, 58, 67^, the health system unit where the innovation will be integrated (i.e. organization, clinic, hospital, community, province etc.) ^11, 15, 17, 22– 25, 27, 28, 31, 33, 34, 37, 39, 43, 47, 48, 53, 56– 58, 67^, and the regulatory, political and economic environment ^12, 13, 15, 17– 19, 22, 24, 25, 28, 34, 39, 40, 43, 48, 52, 53, 55, 57, 58, 61, 67, 68, 72– 74^. These contingency factors are grouped into two themes including stakeholders and context factors, and are used as the headings of the columns of the NTT grid ( Figure 1 and Box 1).

Box 1. **Definitions for the main collaborator groups and contingency factor themes used in the NTT**

**COLUMNS:**

**Researchers (Innovators):** Individuals involved in developing the innovation.

**Practice Community and Innovation Users (End Users):** Individuals from the “health system unit” who will use the innovation (whether that is a health professional, administrator or patient).

**Government and non-government Policy Makers (Decision Makers):** Individuals with policy jurisdiction with the end “health system unit”.

**Physical and Social Environment:** The broader physical and social environment where the innovation will be implemented/scaled up.

**Health System (unit):** Where the innovation will be integrated (organization, hospital, community, system etc.).

**Regulatory, Legislative and Economic Environment:** The broader political and economic landscape.

**ROWS:**

**Pilot Test:** Small scale preliminary study conducted in order to evaluate questions such as the general characteristics of the innovation, cost, and potential impact and capacity to improve health outcomes.

**Implement:** Is the process of putting an innovation into practice in such a way that it meets the necessary standards to achieve the innovations desired outcomes within specific settings. The implementation phase in the tool assumes that the innovation will be implemented in a single setting, or among sites that are contextually homogeneous.

**Scale Up:** Describes an increase in the coverage of health innovations, to both populations that are contextually similar (expand) and diverse (extend), that have been tested in order to benefit more people at a large, national, or international scale.

**Institutionalize:** The sustainable integration of the innovation into existing health systems as a part of their regular service delivery.

### Using the Nose to Tail Tool

**Figure 2.**
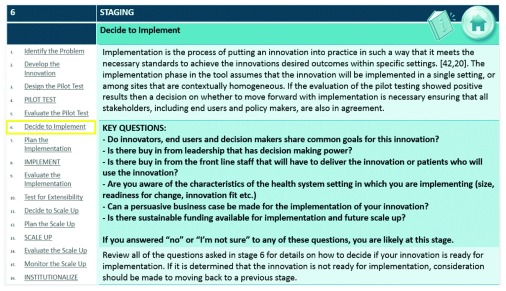
Example of the types of questions asked at stage 6 ( *decide to implement*) to help users of the NTT determine if they are at this stage.

The NTT is designed such that the user’s first task is to determine which of the sixteen stages best represents the current level of maturity of the innovation. The characteristics of the innovation, along with evaluation results, for each stage are described as a standard against which users can gauge where their innovation is in the process. Figure 2 provides an example of the staging guideline used to determine if the user is at stage 6 ( *decide to implement*).

We suggest that all stakeholders (innovators, end users, and decision makers) deliberate together to determine which stage they are at; and that they should start by reviewing the definitions from stage 1 and move forward from there, stopping when they feel they have reached a stage that is not yet completed. This first incomplete stage they reach is the stage their innovation is at.

**Figure 3.**
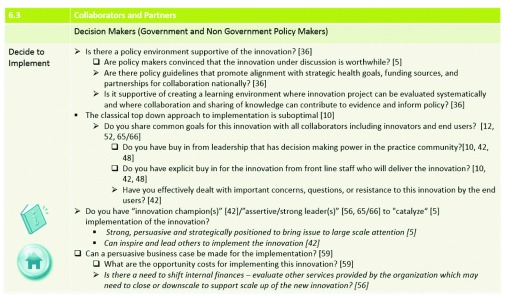
Example of the types of questions the NTT asks decision makers to consider at stage 6 ( *decide to implement*).

The second task is for the users to move across the grid for that row, and review, column by column, the specific considerations for that stage, based on the 6 themes represented in the columns (3 stakeholder perspectives and 3 context factors). The questions, prompts and considerations in each cell are extracted from the reviewed literature. Users should review the questions and prompts to identify potential gaps in their innovation process and to bring attention to factors that may not have been considered sufficiently. Discussion among different stakeholders facilitates consensus on what response or development is required to move forward. Figure 3 provides an example of the types of questions decision makers should consider at stage 6 ( *decide to implement* stage). Of note, in the NTT, action stages (4, 8, 13, and 16) do not have any questions associated with them, as the relevant questions are all considered in the planning (3, 7, and 12) or evaluation stages (5, 9, and 14) preceding and proceeding the action. Evaluation suggestions are embedded throughout the innovation process and formally included after each of the main action stages, highlighting the recognition in the literature of the importance of implementing and scaling up innovations with demonstrated effectiveness and discouraging forward movement, or promoting redevelopment, of those that do not achieve the desired results.

The tool is meant to be used by innovation teams collectively and as such, the questions associated with each stakeholder perspective are directed to that specific stakeholder group (for example, questions under end users are directed to and should be answered primarily by end users who are involved in the innovation development process). However, users of the tool are encouraged to think of the innovation process from all of the stakeholder perspectives and thus consider the questions in each of the columns, as opposed to their own column only, particularly if your team does not have key players from each of these three groups. The questions asked under the context themes are directed to all three stakeholder groups’ collectively.

Although the NTT model appears to follow a simple, single linear path, it is anticipated that not all innovations follow this trajectory. The NTT is built such that users may enter the model at any stage, may go forwards or backwards through the stages, or may skip stages all together. The NTT is meant to be a guide to thoughtful deliberation of the innovation process and not a rigid doctrine.

The NTT grid is available at: http://nosetotailtool.org/. To access this tool, please follow the instructions available at: http://nosetotailtool.org/consent/. In brief, following registration users are provided with the password required to access the tool automatically via email.

### Comparison of the NTT to other frameworks and tools

We compared the NTT to each existing framework or implementation tool found in our search to ensure that the NTT was not duplicative. In our review we found 11 papers describing 7 different frameworks/models which defined one or more stages to the process of innovation, along with contextual factors. None of the frameworks reviewed described more than 7 of the NTT stages (stages 7–14 in Yamey’s Framework for Success in Scaling Up ^15^) while others described as few as 3 stages (stages 7–9 in the QIF ^23^). Individual non-framework papers touched on one or more stages, with 4 papers describing stage 16, institutionalization ^12, 13, 42, 47^, a stage not covered in any of the frameworks reviewed. With respect to the context columns, only one pre-existing framework (CFIR ^25^) considered all of the contextual themes considered in the NTT. Although many frameworks described the importance of working with collaborators, only one (QIF ^23^) developed their framework to be used by all three of the stakeholder groups (decision makers, end users, and innovators). We concluded that the NTT tool offers a more comprehensive and thus potentially useful approach to innovation in healthcare. The Supplementary material provides a descriptive comparison of the NTT in contrast to the five frameworks and two tools we found. A summary of the comparisons can be found in Table 1.

**Table 1.** An overview comparison of the NTT in contrast to five frameworks and two tools found in the scoping review.

	Staging	Context Considerations	Focus	Intended Audience of Framework or Tool	Tool
**NTT**	1–16	Social and Physical Environment; Health System; Regulatory, Legislative and Economic Environment	Pilot Testing Implementation Scale Up	Innovators Decision Makers End Users	Yes
PARIHS*	NTT 5–9	Health System	Implementation	Innovators	Yes
**CFIR**	NTT 2–9	Social and Physical Environment; Health System; Regulatory, Legislative and Economic Environment	Pilot Testing Implementation	Innovators	No
**T0-T4**	NTT 1–9	Social Environment; Health System; Economic Environment	Pilot Testing Implementation	Innovators	No
**QIF**	NTT 7–9	Social and Physical Environment; Health System	Implementation	Innovators Decision Makers End Users	Yes
**Framework for** **Success in Scaling Up**	NTT 7–14	Health System; Regulatory and Legislative Environment	Implementation Scale Up	Innovators	No
**AIDED**	NTT 10–14	Social and Physical Environment; Health System; Regulatory, Legislative and Economic Environment	Scale Up	Innovators	No
**Conceptual Model of** **EBP Implementation**	NTT 1–2, 4, 6–9, 15	Social Environment; Health System; Regulatory, Legislative and Economic Environment	Implementation	Innovators	No

*PARIHS – Promoting Action on Research Implementation in Health Services (Stetler)*

*PARIHS was orginally developed by Kitson *et al.* in 1998 and was revised by Stetler in 2011. This review looked at Stetler’s revised version.

*CFIR – Consilidated Framework for Implementation Research (Damschroder)*

*T0 – T4 - Glasgow’s 5 Key phases in moving Research to Practice/Policy (Glasgow)*

*QIF – Quality Implementation Framework (Meyers)*

*Framework for Success in Scaling Up (Yamey)*

*AIDED (Perez-Escamillla)*

*Conceptual Model of Evidenced-Based Practice Implementaiton (Aarons)*

## Discussion

### A collaborative and deliberative decision making tool

A striking, yet common theme in much of the literature was the need for improved collaborations among key stakeholders, which we identified as innovators, end users and decision makers. Deliberation is more than just a discussion of issues; it is collective “problem solving” that allows “individuals with different backgrounds, interests and values to listen, understand, potentially persuade and ultimately come to more reasoned and informed decisions” ^75^.

The NTT has the potential to start the process of closing the gap between research and policy. Despite 40 years of attempting to translate research into evidenced based policy, barriers continue to persist ^76^. Ellen *et al.* conducted a qualitative study to identify barriers and facilitators for implementing supports for evidenced informed decision making and highlights three main areas: facilitating pull efforts, establishing a climate for research use, and linkage and exchange ^77^. Pull efforts include implementing technical infrastructures that allow “easy access” to research through physical tools; and linkage and exchange efforts which ensure that “decision makers have the necessary skills and connections to acquire, assess, adapt and apply the necessary evidence” to decision making ^77^. From these findings however, it is evident that the authors assume that problems identified by decision makers have existing solutions or answers and the challenge is simply in finding them. We challenge that this is often not the case, and that the healthcare system is too complex to simply “join [found] solutions to problems” ^76^. There is a process required for solving healthcare problems and that requires all stakeholders to work collaboratively from the onset and not just at the point of implementation or scale up. The NTT proposes that decision makers, end users and innovators be involved from the very beginning, the point of identifying problems in the healthcare system and remain involved throughout the development of the innovation. This allows mutual exchange of information throughout the process; it allows decision makers to discuss with innovators at the onset whether they feel that the problem at hand is a priority that needs to be solved and therefore will have support for it; it allows decision makers and end users to provide input into the design of the innovation, how it’s pilot tested, and can highlight which outcomes are important for them to support moving forward with the project. It ensures that decision makers, end users and innovators share common goals throughout the process. The NTT facilitates the potential to co-create and co-produce knowledge, developing a bridge between research and policy, which allows for a more democratic and useful knowledge exchange ^76^.

The NTT also emphasizes the importance of collaborative decision making with end users. The field of co-creation and design is an evolving field that was born from the merging of user-centred design (“user as subject”) and the participatory approach (“user as partner”) ^78^. Co-design and creation can broadly be defined as the creativity of designers (innovators) and people not trained in design (end users and decision makers) working together in the development process ^78^. Healthcare innovators need to start embracing the attitudes that have led to success in the private business sector; “we believe the key ingredient of innovation is to provide a compelling experience to all participants based on network effects for value creation… a platform of innovation for convergence of expertise/ideas, collaboration among participating organizations, and co-creation of the shared value with customers should be the core of co-innovation” ^79^. Integrating users in the early stages of the development process can have impacts with positive, long range consequences” ^78^. Integration and collaboration throughout the innovation process at all key moments of decision making is believed to be the missing ingredient needed for sustainable solutions.

### Comprehensiveness of the NTT

The NTT covers the innovation process from problem identification through to institutionalization, where the innovation becomes integrated into common practice. It was intentionally designed to provide a single tool covering the entire process of innovation, from the beginning to end, hence the name, Nose to Tail. The NTT prompts consideration of the most important contextual barriers, categorized in broad domains: social and physical environment, regulatory and economic considerations and health system context. Although several of the models take into consideration some of these contextual domains, only one other, the CFIR ^25^, takes into consideration all of these, and none groups them in a way that optimizes discussion among stakeholders, as the NTT does. This comprehensiveness is intended to mimic the real world process of innovation, and allow users to assess success or delay in innovations at any stage of their lifecycle; only a comprehensive view from beginning to end allows all successes and failures to be identified. This has a practical value as it improves the continuity of the discussions between stakeholders on a given innovation.

As a tool, the NTT is intended for iterative use, by the deliberating stakeholders, alone and together, at or before each stage of the process. The NTT creates a grid which connects the stages of innovation to the relevant contextual issues, and at each stage identifies the specific concerns that might arise at that stage in relation to each of the contextual domains. This allows a stage specific, and thus more focussed, discussion on what barriers may require adaptations to the innovation, rather than a broad and generalized discussion of barriers, unconnected to the current stage of development of the innovation.

The NTT is designed to be deliberative and thus preventative, focussing discussion between multiple stakeholders, including health innovators, decision makers and end users on potential barriers to scale up as they come into view, allowing for innovations to be sequentially adapted before meeting these problems in the “real world” setting. The use of the tool is meant to practically support an innovation from the stages of development through to sustainment, or alternatively to propose the appropriate discarding of unadaptable, unacceptable, or ineffective innovations, whereby one or more stakeholder group finds an insurmountable barrier to supporting further development or implementation of the innovation.

In addition to being comprehensive in covering the entire process of innovation, the NTT is also all-inclusive in that it can support health innovation development in any healthcare system setting, whether it be in a LMIC or high income country (HIC). Over half of the literature reviewed comes from the LMIC setting. Examples of the entire innovation process, from idea to institutionalization, are more likely to be seen in LMICs given their distinctive characteristics that provide powerful incentives, or “gaps” that drive innovation ^80^. These gaps include: (1) a performance gap that requires higher volumes of satisfactory performing innovations for lower prices boosting development low-cost innovations of acceptable quality; (2) an infrastructure gap that provides a “clean slate” where building and implementing from scratch eliminates the need to overcome existing infrastructure barriers; (3) a sustainability gap that emphasizes development of “green” solutions that will not deplete existing natural resources or cause further damage in settings with large populations; (4) a regulatory gap that reduces policy barriers on implementation and scale up of innovations; (5) and a preference gap where unique preferences from different populations promote creativity in design ^80, 81^. In the context of health care, overwhelming need adds motive to create effective solutions that are scalable ^80^. These gaps describe why reverse innovation (RI) is possible; the process of first identifying or fostering a successful innovation in the LIC that addresses an unmet need in a HIC ^80, 81^. The NTT is consistent with RI, highlighting that lessons around innovation development can be learned from LMICs and applied to HICs; even if expenditures are different, facilitators and barriers are common in all health systems.

### The role of implementation theory in the innovation process

The NTT attempts to address the need raised by Colquhoun for a shared and overarching approach that could promote effective communication between all stakeholders ^3^. It does so, not by creating a common language for discussing behavioural, organizational or other social sciences theories among researchers in the knowledge translation, dissemination and implementation communities, but by creating a simple scaffolding for deliberative discussion among stakeholders involved in developing, implementing and scaling up a given innovation. Its intent is practical, not theoretical ^5, 82^.

Although the argument has been made that a shift towards theoretically driven implementation interventions is necessary ^83^, the choice to be atheoretical was purposeful. The ICEBeRG authors assert that through the use of explicit behavioural theories that produce quantifiable results in implementation research, researchers will be better able to identify predictors of success that are common across different contexts; and should thus use these predictors to design interventions which may be more widely applicable ^83^. We argue that the plethora of overlapping and contradicting theories makes it difficult to judge the applicability of a piece of empirical evidence in supporting one theory over another; and from a practical viewpoint argue that the usefulness of the theoretical enterprise is undermined by the challenge in designing interventions which closely match only one among several overlapping theories ^82^. Oxman states that is time for us to “work collaboratively, based on common sense (sound practical judgment that is independent of specialized knowledge or training), sound logic and rigorous evidence to help people make informed choices about health care ^5^. The NTT is an attempt to organize published opinion and empirical experience for this purpose.

The NTT tool could also, if the reader prefers, be considered to be an implicit, mid-range “theory” ^83, 84^ along the following rather common sense lines:

1. Innovations progress through stages on route to scale up or failure to scale.
2. Progress through these stages is contingent on overcoming hurdles through adaptation of the innovation.
3. The hurdles are related to
    a) features of the innovation itself;
    b) the broader context in which the innovation is being implemented or scaled; and
    c) support for development and scale up of the innovation from all stakeholders.

This “theory” gives rise to an intervention hypothesis: the process of developing a shared understanding of the different stakeholders’ perspectives through discussion improves adaptation and progress of an innovation through the stages outlined in the NTT (or leads to appropriate abandonment at an early stage).

### Limitations

We have derived each of the NTT stages and the contents of each cell’s prompts and issues for consideration from the literature that we reviewed. In that sense, this tool is evidence based rather than theoretical. Admittedly, many of the included papers are themselves not empirical; and even those that are descriptions of instances of innovation, at one or more stages, are not necessarily methodologically excellent, neither in qualitative nor quantitative terms. So in this sense, the NTT is taking as its raw material, a set of opinions and described experiences, and organizing these into patterns for ease of use. We find the simplicity and coherence of the grid to be attractive.

This scoping review which led to the development of the NTT used “scale up” as the central term. It was selected given the widespread agreement that many effective interventions exist to address many of the health problems but fail to be effectively implemented or scaled to sustainment. While further searching, especially using a less specific and wider set of terms e.g., “dissemination” would undoubtedly increase the number of retrieved articles enormously, it is not clear to us that this wider search would improve on the framework of stages and domains which we have assembled and for this reason we elected to move forward with our smaller range of papers now, but leave open the door for further inputs and we do not exclude the possibility of a systematic review in the future.

The NTT is not attempting to replace any of the competing theoretical frameworks, which will presumably strengthen over time, as evidence for one or another framework, or aspects of a framework, accumulates from empirical studies of innovation processes ^83^. The NTT could be used to collect data on a large number of innovations, and the descriptive and analytic epidemiology of these instances of innovation could contribute to an empirical evidence base for these theories.

### Summary and next steps

Using a brief, sensitive and specific search strategy we have identified and abstracted information from 69 published papers describing empirical instances of scale up or descriptions of frameworks for understanding or planning parts of scale up. This scoping review is only the first stage of data gathering for this tool, and was not intended to be comprehensive, but has nevertheless given us sufficient information to compose a grid of 16 distinct and well defined stages and 6 distinct and well defined contextual domains relevant to the progress of an innovation in healthcare from problem identification to sustained solution. This relatively small number of included papers allowed us to reach initial conceptual saturation, which we defined for each cell (the intersection of a stage and contextual domain) as having one or more relevant issues for deliberation between stakeholders. The number of papers contributing to each cell in the table was obviously often much smaller than that supporting most of the stages or domains and so it seems that increasing the deliberation material for each cell warrants further searching, We propose a new approach for obtaining information on issues that should be considered for each cell, namely crowdsourcing, to contribute this added detail. Crowdsourcing can be simply defined as the posting of a problem online whereby a large number of individuals have the opportunity to offer solutions to the problem ^85^. Alongside publication of this paper, we have set up a website ( nosetotailtool.org) containing the grid, with a straightforward process by which any member of the stakeholder communities can provide comments and feedback on any of the stages, domains or individual cells which we will use to improve the tool. We seek readers’ comments, preferably with specific citations to the literature or brief factual descriptions of their experiences. We will never quote you without permission, never use your information or email details other than to contact you to discuss your comment or request permission to quote you, we will delete your personal information every 24 months, and will at all times store your details in encrypted format.

At this time, we believe the NTT 1.0 is a minimally viable product (MVP) ^29^ (positioned in stage 2 of the NTT, “Develop the Innovation”) that will evolve over time and we strongly believe that the underlying evidence base will strengthen over time. An MVP is an early prototype of the innovation that is typically deployed to as subset of possible customers, such as early adopters that are more likely to give feedback and able to grasp the innovation vision and hypothesis ^29^. It is the version of the innovation which allows the team to collect validated learning about from users before large investments are made in its development ^29^.

In addition to crowdsourcing we are currently conducting “hypothesis testing” ^29^ using our prototype with healthcare innovation teams within Ontario, Canada and preliminary feedback has been positive. From this testing we have seen that the stages in the tool seem to match the users (innovators) perceptions of stages they have gone through and that the questions asked at each stage expose assumptions requiring further deliberation.

In addition to this, we are investigating some new uses to the NTT (in our terminology, extensions): we are working with healthcare funders to assess the value of the tool in innovation portfolio analysis, whereby the tool could provide an overview of the progress of a portfolio of innovations for which they are currently funding; and, although the tool already emphasizes the importance of evaluation at each stage, we are working on a review to determine what patterns or sequences of evaluation designs best support advancement of the innovation at each stage.

## Conclusion

There is a mismatch between good science and the complexity of health systems. Even if you have a good idea and a good innovation that is supported by empirical science that is simply not enough; the health system is complex and good innovations alone will not necessarily be scaled in real world settings. Successful development, implementation and scale up of health innovations is a multi-stage process that requires appraisal at every stage and it is a team effort that requires true collaborations from all stakeholders at every stage. It is essential to be constantly aware of what stage the innovation is at and to identify what contextual barriers require overcoming before moving forward in the process. At present, innovations are commonly rushed through stages and even skip essential stages all together; innovations are implemented or scaled up prematurely without evaluations to verify that they are mature enough to advance forward.

The NTT tool is an atheoretical, stage based and context aware tool that helps innovators, decision makers and end users identify in a deliberative and potentially collaborative fashion, what they have done to get the innovation to its current stage and identify what needs to be done to move it forward successfully. The NTT tool is meant to be a guide to iterative deliberation through the innovation process. This tool emphasizes the need to identify barriers early and repeatedly at each stage in the innovation process. The tool may suggest a need to go back to earlier stages and re-design the innovation, or in some cases to abandon the project all together.

The NTT tool is a comprehensive and consistent way of thinking of the entire innovation process. We believe that if the end goal of widespread jurisdictional scale up and sustainment of appropriately chosen and carefully adapted innovations is kept in mind from the beginning, success is more likely.

## Data availability

The data referenced by this article are under copyright with the following copyright statement: Copyright: © 2016 Gupta A et al.

Data associated with the article are available under the terms of the Creative Commons Zero "No rights reserved" data waiver (CC0 1.0 Public domain dedication). 

*F1000Research*: Dataset 1. NTT scoping review. Includes the initial information collected from the initial 63 articles reviewed which were used to determine the stages (rows) and contingency factors (columns) for the NTT, 10.5256/f1000research.8145.d115651 ^86^

## Supplementary material

A descriptive comparison of the NTT in contrast to the five frameworks and two tools found in the scoping review.
