# Supplementary material for: Promoting development and uptake of health innovations: The Nose to Tail Tool
Source: F1000Res. 2016 Mar 16;5:361. [Version 1] doi: 10.12688/f1000research.8145.1 (PMC4863676; doi:10.12688/f1000research.8145.1)
Supplement: Supplementary file 2 [file f1000research-5-8761-s0001.tgz › c658ec64-894f-4f20-80ed-1b0ab8bbb1f4.docx]

1. *Promoting Action on Research Implementation in Health Services (PARIHS)*

PARIHS was developed by Kitson^1^ and colleagues in 1998 and was revised by Stetler el al. in 2011^2^. The PARIHS framework looks specifically at supporting successful implementation of interventions supported by empirical research ^3^ and coincides with NTT stages 5 through 9. It believes that successful implementation is a function of the evidence for the intervention and the facilitation of implementation ^2^. In the NTT, facilitation of implementation is considered in both the innovators and the health system columns. PARIHS considers contextual factors in the NTT’s health systems column but does not distinguish between environmental, regulatory or economic contextual factors.

PARIHS focuses on two forms of implementation: first, interventions with a task oriented change being implemented in a short time frame; or second, interventions with an organizational purpose such as implementation of strategies with the aim of instilling transformational change at one or more levels of an institution ^2^.

PARIHS is similar to the NTT in that it also offers a tool to support implementation of evidenced based innovations into practice ^2^. The PARIHS tool’s intended audience is researchers and innovators as it aims to support new investigators looking for “theoretical assistance” ^2^. This is in contrast to the NTT, which is a deliberative and atheoretical tool which aims to provide structure to deliberations between stakeholders on adaptations needed, as an innovation progresses along its path.

1. *Consolidated Framework for Implementation Research (CFIR)*

The CFIR looks only at the contextual factors that influence successful implementation ^4^. It considers factors that influence innovation development through to evaluation of implementation coinciding with NTT stages 2 to 9. The contextual factors in the CFIR are similar to the ones considered in the NTT; CFIR considers the intervention characteristics (considered in NTT staging and innovator columns), outer setting (parallel to NTT regulatory, legislative and economic context and social and physical environmental contexts), inner setting (considered in NTT health system context), characteristics of individuals (considered in NTT health system context) and processes (overlap between rows and column considerations in NTT). The CFIR was designed primarily for use by researchers and innovators as opposed to decision makers and end users ^4^ and is not designed as a tool to assist in the development or implementation of an innovation.

1. *Glasgow’s 5 key phases in moving Research to Practice/Policy (T0-T4)*

Glasgow’s work looks specifically at how to close the gap between interventions with demonstrable benefits and its implementation into clinical practice ^5^. He describes 5 stages (T0-T4) which start at problem identification and end at evaluation of the implementation ^5^, which coincide with NTT stages 1-9. This model does not specifically consider scale up of the innovation or its sustainment thereafter (although there is no precise definition of implementation, and so this could be considered to take place at any scale between a single facility to multiple jurisdictions). Like the NTT, Glasgow’s model highlights the importance of taking into consideration contextual factors such as the financial and political environment and the staff and organization of the health system delivering the innovation. Although it does not explicitly discuss all of the contextual factors considered in the NTT, it does highlight that the intention is to promote approaches that allow for successful implementation in “real-world” settings ^5^ which coincides with the overall goal of the NTT.

1. *Quality Implementation Framework (QIF)*

The QIF outlines 14 action steps to foster high quality implementation which broadly encompass four phases including initial considerations regarding host setting (considered in NTT context columns), creating a structure for implementation (parallel to NTT stage 7), ongoing structure once implementation begins (considered in NTT stages 7 and 9) , and finally improving future applications (considered in NTT stage 9) ^6^. The QIF is also designed as a tool, the Quality Improvement Tool (QIT), which is in the format of a worksheet that can be used by implementation teams ^6^. The QIF tool is similar to the NTT in that its intended users include innovators, decision makers and end users.

1. *Framework for Success in Scaling Up*

Yamey’s proposed framework for successful scale up places the emphasis on six different categories including the attributes of the innovation (considered in NTT staging and innovator columns), attributes of the implementers (considered in NTT innovator and decision maker columns), the chosen delivery strategy (considered at NTT stages 10 - 14), attributes of the adopting community (considered in the NTT social environmental and health system context), the sociopolitical context (considered in the NTT regulatory and economic environment), and the research context (considered in the NTT innovator column)^7^. Yamey’s proposed framework is primarily targeted for use by innovators.

1. AIDED Framework

AIDED was designed to be an integrated and practical framework for scale up^8^ (considered in NTT 10 -14). It proposes that there are five nonlinear but interrelated components that contribute to successful scale up including: assessing landscape (considered in NTT context columns), innovating to fit user receptivity (considered in NTT staging column), developing support, engaging user groups and devolving efforts for spreading the innovation (considered in NTT stakeholder columns). The AIDED framework’s focus is on adapting and integrating innovations to fit new contexts when scaling up. The AIDED framework is primarily targeted for use by innovators, however does emphasize the importance of collaborating with end users and decision makers.

1. Conceptual Model of Evidenced Based Implementation

Aarons et al presents a conceptual framework that integrates high yield variables felt to play a role in achieving effective implementation of evidenced based practices^9^. They propose that there are four key implementation phases including exploration (parallel to NTT 1-2), adoption/preparation (parallel to NTT 4, 6-7) , active implementation (parallel to NTT 8) and sustainment (parallel to NTT 9 and 15). At each of these phases they describe several “outer context” (considered in NTT context columns including social, regulative and legislative factors) and economic” and “inner context” (considered in the NTT health system context column) factors that may impact the implementation differently depending on what phase you are at.
