# Supplementary material for: Promoting development and uptake of health innovations: The Nose to Tail Tool
Source: F1000Res. 2016 Mar 16;5:361. [Version 1] doi: 10.12688/f1000research.8145.1 (PMC4863676; doi:10.12688/f1000research.8145.1)
Supplement: Peer review history

# Peer review: Referee response for version 1

**DOI:** 10.5256/f1000research.8761.r14091

**Published:** 2016-06-01

**Authors:** Nerges Mistry

**Affiliations:** 1 Director and Trustee, Foundation for Research in Community Health, Mumbai, India

The article is extremely useful  for intervention and operations researchers who examine a systemic innovation, have a successful proof of concept studies and attempt to upscale simple or complex interventions on a national or even a regional scale.

 The paper aims to detangle the thought jungle that accompanies this effort and give a procedural element to this effort. This detangling itself is a laudable effort and deserves to be indexed but it maybe an effort that is fraught with deviations in various parts of the world with different dynamics between innovators, end users and the decision making environment. It would be nice for the authors to identify those willing to use the NTT in different parts of the world to guage its utility in different scenarios. Within countries also end users maybe heterogenous so the limits of scalability in a particular setting need to be contextualized. It would be interesting to see articles on NTT use in a variety of innovations  a couple of years from now.

 Secondly as soon as an innovation is framed, a concept which seems to be missed out in the NTT is the gauging of its life span. This requires information on the evolution stage of the condition for which the innovation is planned as also the stage of parallel competitive technologies or approaches  that are being worked on to obtain the same or similar output. Perhaps this is one sort of additionality that maybe inserted in Fig 1 .

 The NTT would serve as a valuable checklist for both simple and complex innovations and looks promising as a reliable guidance tool. However the proof of the pudding is in the eating for which a platform may need to be consciously created to get views on its utility and the omissions.

I have read this submission. I believe that I have an appropriate level of expertise to confirm that it is of an acceptable scientific standard.

# Peer review: Referee response for version 1

**DOI:** 10.5256/f1000research.8761.r13537

**Published:** 2016-05-20

**Authors:** Saravana Kumar

**Affiliations:** 1 Division of Health Sciences, University of South Australia, Adelaide, Australia

Thank you for asking me to review this manuscript. Overall, I was pleased to read this manuscript which outlines the development of a unique tool called Nose to Tail Tool (NTT). It is innovative and in an area which has so much theoretical science (evidence implementation/implementing change) it was refreshing to read about process which resulted in the development of a practical tool that could be utilised by a range of health care stakeholders. I commend the authors for extensively reviewing the literature (although the methodology is a bit weak) and placing the NNT in comparison to other frameworks in this space.

 While there is a lot to like about this manuscript, there are some issues to consider too. They are:

1. The methodology – while I understand that this is a scoping review and as such a formal critical appraisal was not undertaken, I was surprised to note that the searching was confined to PubMed only. Why was this the case? PubMed is quite rudimentary and given the amount of work that has been undertaken, this could have extended to other mainstream databases too (such as Cinahl, Embase, Medline etc). I also would have liked a bit more information about the developmental process. For example, the authors state that “Each of the three categorizations (stages, themes and contingency factors) went through three iterations.” What happened during those stages? How did the authors respond to the findings during those stages?
2. The burden of complexity – While the NNT is quite detailed, I worry if it will also be its Achilles Heel. I suspect these tools are aimed at those at the coal face and given the complexity of the tool (I understand the reasons underpinning it), I worry many clinicians could be put off by it. A tool needs to have good clinical utility and I suspect while this may be useful for research and/or evaluation purposes, how readily and effectively clinicians use this remains to be seen. I will be interested to know about the pilot study findings.
3. The manuscript itself – I must say the manuscript is very long and verbose! While I am aware that *F1000Research *may not have a word limit, it is the duty of the researchers to ensure what is presented is reader-friendly and engaging. Given that most manuscripts are 4000 words (approx.), I think this would be double that word count. As it stands, this manuscript could be trimmed down to make it more succinct and punchy.

I have read this submission. I believe that I have an appropriate level of expertise to confirm that it is of an acceptable scientific standard, however I have significant reservations, as outlined above.

# Peer review: Referee response for version 1

**DOI:** 10.5256/f1000research.8761.r13642

**Published:** 2016-05-10

**Authors:** Jeffrey Braithwaite

**Affiliations:** 1 Centre for Healthcare Resilience and Implementation Science, Australian Institute of Health Innovation, Macquarie University, Sydney, Australia

This paper represents a very useful development. The authors have painstakingly reviewed and synthesised the articles useful for scaling up interventions (specifically, innovations) and created a model for innovators to follow, or check their progress. This kind of staged approach is useful for those who follow logical, structured ways of thinking and working.

My sole reservation is that models such as this downplay the point that health care is a complex adaptive system. In the real world, people are busy, challenged, and stressed, for the most part, and rarely use tools and frameworks in a logical, structured manner. And innovations rarely unfold in neat and tidy ways. I would like to see some recognition of this in papers such as this. Perhaps the authors in a future piece of work could factor in the complexities, and the fact that most clinicians flex and adjust their practices to meet exigencies as the real world unfurls before them.

I have read this submission. I believe that I have an appropriate level of expertise to confirm that it is of an acceptable scientific standard.

# Peer review: Referee response for version 1

**DOI:** 10.5256/f1000research.8761.r13086

**Published:** 2016-03-29

**Authors:** Elizabeth Molyneux

**Affiliations:** 1 Department of Paediatrics, University of Malawi, College of Medicine, Blantyre 3, Malawi

Much innovation research is all about developing an innovation, with too little attention paid to how the new tool or idea will be, or can be, implemented. Gupta *et al.* have done a literature search of the subject to review how innovators plan (or not) for the whole process from development and funding through to implementation and commercialisation. They have developed a planning programme called the Nose to Tail Tool which provides steps for action and steps for deliberation, by all the players concerned along the entire process of innovation implementation. Specific questions are raised with different groups of people during the course of development. The NTT tool is a planning grid (of 72 boxes) which helps bring everyone together early in the development phase so that problems further down the line can be anticipated or avoided. Discussions may even lead the team to decide not to waste further time and effort on the project.

Gupta states that the tool is being put to use in Canada; it will be interesting to see how effective it is; experience with using the tool will probably lead to some fine-tuning.

I like the idea of the NTT. I think the paper is long, with quite a bit of repetition; but it is worth a read and the NTT could save innovators both time and money.  But the proof of the pudding will be in its successful use.

I have read this submission. I believe that I have an appropriate level of expertise to confirm that it is of an acceptable scientific standard.
